# Occupational health factors associated with work ability among early childhood education teachers

**DOI:** 10.3389/fpubh.2026.1847583

**Published:** 2026-09-01

**Authors:** Ivana Nikolić, Snježana Mraković, Marko Badrić

**Affiliations:** Faculty of Teacher Education, University of Zagreb, Zagreb, Croatia

**Keywords:** early childhood education teachers, musculoskeletal pain, occupational health, physical activity, work ability, work-related functional limitations, work-related stressors

## Abstract

**Background:**

Musculoskeletal disorders and psychosocial stressors are highly prevalent among early childhood education (ECE) teachers and may adversely affect work ability. This study examined occupational health factors associated with work ability among Croatian ECE teachers, with particular focus on musculoskeletal pain, work-related functional limitations, and exposure to work-related stressors.

**Methods:**

A cross-sectional online survey was completed by 472 early childhood education (ECE) teachers. Measures included musculoskeletal pain across six body regions, work-related functional limitations, work-related stressors, physical activity, and an adapted single-item measure of current work ability. Hierarchical regression analyses were used to examine occupational health factors associated with work ability, and mediation analyses (PROCESS, 5,000 bootstrap samples) were conducted to examine indirect associations.

**Results:**

Work-related functional limitations and exposure to work-related stressors showed the strongest negative associations with work ability. Musculoskeletal pain was not independently associated with work ability in the final regression model, but mediation analyses were statistically consistent with indirect associations via functional limitations and work-related stressors. Teachers who met WHO physical activity recommendations reported slightly higher work ability and more favorable occupational health indicators, although physical activity was not independently associated with work ability.

**Conclusion:**

Work ability among early childhood education (ECE) teachers was most closely associated with work-related functional limitations and work-related stressors. Musculoskeletal pain was not independently associated with work ability, although the mediation analyses were statistically consistent with indirect associations via functional limitations and work-related stressors. These findings highlight the importance of considering both physical and psychosocial aspects of occupational health when addressing work ability in ECE settings. Given the cross-sectional design, the observed associations should not be interpreted as causal.

## 1 Introduction

Work ability is a central indicator of occupational functioning and reflects the balance between job demands and individual resources (1). It is particularly relevant in professions characterized by substantial physical and psychosocial demands, as reduced work ability has been associated with increased sickness absence, work disability, and early exit from the workforce. Although the Work Ability Index is the most widely used instrument for assessing work ability, single-item measures have demonstrated strong predictive validity for work disability and sick leave (2), making them suitable for large-scale occupational health research.

Early childhood education (ECE) teachers are exposed to a range of occupational health factors that may influence work ability. Their daily work involves lifting and carrying children, frequent bending and squatting, kneeling, prolonged standing, and performing activities using furniture that is not adapted to adult anthropometric characteristics. These demands contribute to a high prevalence of musculoskeletal pain and musculoskeletal disorders, particularly in the lower back, neck, and upper limbs (3). Previous studies have consistently identified lifting, awkward postures, and ergonomic strain as important correlates of musculoskeletal symptoms (4, 5), while longer professional tenure has been associated with an increased likelihood of musculoskeletal pain, suggesting cumulative load effects across years of work (6).

In addition to physical demands, ECE teachers experience considerable psychosocial pressures. Emotional labor, responsibility for children's safety, administrative requirements, and work with children with diverse needs contribute to occupational stress (7). Within the Job Demands–Resources framework (8), high job demands combined with limited resources increase the likelihood of strain, burnout, and reduced work ability. Research in educational and care settings indicates that physical and psychosocial demands jointly contribute to musculoskeletal symptoms and reduced work capacity more strongly than either domain alone (9), whereas resources such as supportive supervision and collegial relationships may mitigate these adverse effects (10, 11).

Existing research indicates that musculoskeletal pain, work-related functional limitations, and psychosocial stressors are closely interconnected. Psychosocial strain has been associated with musculoskeletal complaints (12), while work-related functional limitations have been identified as a potential pathway linking pain to work-related outcomes in physically and organisationally demanding occupations (13). Although multiple international studies have documented musculoskeletal pain and occupational health challenges among ECE teachers, research integrating physical and psychosocial factors associated with work ability within a single analytical framework remains limited. To our knowledge, no studies have simultaneously examined these factors among Croatian ECE teachers.

The aim of this study was to examine the associations between musculoskeletal pain, work-related functional limitations, work-related stressors, physical activity, and self-rated work ability among Croatian early childhood education (ECE) teachers. In addition, the study examined whether work-related functional limitations and work-related stressors were statistically consistent with indirect pathways linking musculoskeletal pain and work ability, and whether compliance with World Health Organization (WHO) physical activity guidelines (14) was associated with occupational health indicators and work ability. The specific hypotheses are presented below.

*H1*: Higher levels of musculoskeletal pain, work-related functional limitations, and work-related stressors are statistically associated with lower self-rated work ability among early childhood education teachers.
*H2*: Demographic characteristics (age, educational attainment, kindergarten location, and age group of children) account for a small and statistically non-significant proportion of variance in work ability, whereas the addition of occupational health factors significantly increases the explained variance.
*H3*: Early childhood education teachers who meet the WHO PA guidelines report more favorable health indicators and higher work ability than those who do not meet the guidelines.
*H4*: Work-related functional limitations statistically mediate the association between musculoskeletal pain and work ability.
*H5*: Work-related stressors statistically mediate the association between musculoskeletal pain and work ability.

## 2 Methods

### 2.1 Participants

A total of 472 preschool teachers employed in early childhood education and care (ECEC) institutions across the Republic of Croatia participated in this study (Table 1). The sample included participants from all 20 counties and the City of Zagreb, ensuring broad regional representation and coverage of all administrative areas of the country. The largest proportion of respondents was recruited from the City of Zagreb (31.8%), followed by Osijek-Baranja County (11.0%), Međimurje County (9.7%), and Koprivnica-Križevci County (7.2%), while the remaining counties were represented by smaller proportions.

**Table 1:** Sample characteristics (*N* = 472).

Variable	Category	*N*	%	*M (SD)*
Age (years)	–	—	—	39.3 (11.1)
Work experience (years)	–	—	—	13.3 (11.1)
Gender	Female	467	98.9	—
	Male	5	1.1	—
Educational attainment	Bachelor's degree	255	54.0	—
	Master's degree	144	30.5	—
	Secondary school	26	5.5	—
	Student	32	6.8	—
	Other	15	3.2	—
Kindergarten location	Urban	352	74.6	—
	Rural	120	25.4	—
Age group of children	Nursery group (1–2 years)	114	24.2	—
	Younger age group (3–4 years)	63	13.3	—
	Middle age group (4–5 years)	79	16.7	—
	Older age group (5–6 years)	90	19.1	—
	Mixed-age group	126	26.7	—
WHO PA meets guidelines	Yes	212	44.9	—
	No	260	55.1	—

The sample was highly feminized (98.9% women and 1.1% men), reflecting the gender distribution characteristic of the early childhood education profession. Participants varied in terms of age, years of service, educational level, kindergarten location (urban vs. rural), and the age group of children with whom they worked. Data on compliance with the WHO PA guidelines (14) were also collected and included in the analysis.

The study was conducted in accordance with the Declaration of Helsinki and with the Ethical Code of the University of Zagreb and the Ethical Code for Research Involving Children. The study was approved by the Ethics Committee of the Faculty of Teacher Education, University of Zagreb, Croatia (protocol code 641-03/25-01/12; Ref. No.: 251-378-01-25-2; approval date: 26 November 2025).

### 2.2 Research instruments

Due to the lack of validated instruments specifically designed for preschool teachers in Croatia, an author-developed questionnaire was constructed to assess six constructs: health burden, musculoskeletal pain, work-related functional limitations, work-related stressors, work ability, and PA.

The initial item pool was generated based on relevant conceptual frameworks from ergonomics, occupational kinesiology, work psychology, and pedagogy (8, 14, 15). Content validity was evaluated by an expert panel (kindergarten directors, educational specialists, and preschool teachers), and the instrument was pilot-tested on a sample of 50 participants to assess item clarity and the preliminary factor structure. For all multi-item scales, mean composite scores were calculated, with higher scores indicating greater health burden, more frequent musculoskeletal pain, higher work-related functional limitations, or greater exposure to stressors. Complete item content and response formats for the study-specific scales are provided in Supplementary Tables S1–S4, while item-level communalities and factor loadings for the MSP, WFL, and WRS scales are presented in Supplementary Table S5.

Health Burden (HB) assessed the presence and perceived impact of eight groups of chronic conditions (e.g., hypertension, cardiovascular and respiratory diseases, spinal disorders, thyroid disease, osteoporosis, arthritis, and other rheumatic conditions). Participants rated the extent to which each condition affected their daily life and work on a five-point scale (1 = *not present* to 5 = *affects me greatly*). Exploratory factor analysis supported a one-factor solution (*KMO* = 0.75; Bartlett's χ^2^(36) = 720.25, *p* < 0.001), explaining 32.2% of the total variance, with acceptable internal consistency (Cronbach's α = 0.71).

Musculoskeletal Pain frequency was assessed with the question: “How often have you experienced musculoskeletal pain during the past six months?” across six anatomically defined body regions (neck, shoulders/upper limbs, lower back, hips, knees, and feet), rated on a five-point scale (1 = *never* to 5 = *daily*). Exploratory factor analysis supported a one-factor structure [*KMO* = 0.82; Bartlett's χ^2^(15) = 904.23, *p* < 0.001], accounting for 52.8% of the variance, with good internal consistency (Cronbach's α =0.82).

Work-related Functional Limitations measured the extent to which health problems interfered with typical work tasks during the previous month. Using a five-point scale (1 = *not at all* to 5 = *extremely*), participants evaluated six domains: participation in PA with children, prolonged standing and walking, bending/squatting/lifting, concentration and attention, energy level, and performance of physically demanding pedagogical tasks. Exploratory factor analysis supported a one-factor solution [*KMO* = 0.89; Bartlett's χ^2^(15) = 2033.09, *p* < 0.00], explaining 72.1% of the variance, with excellent internal consistency (Cronbach's α =0.92).

Work-related Stressors were assessed in accordance with the Job Demands–Resources model (8) and captured nine common organizational and interpersonal demands in preschool settings. Participants rated how often they were exposed to each stressor on a five-point scale ranging from 1 (*never*) to 5 (*very often*). The stressors included having too many children in the group, lack of time to complete tasks, administrative pressures, working with children with special needs, relationships with parents, relationships with colleagues, lack of supervisory support, unpredictable situations at work, and limited spatial or material resources. Exploratory factor analysis supported a one-factor solution (*KMO* = 0.88; Bartlett's χ^2^(36) = 1671.25, *p* < 0.001), which explained 49.7% of the variance, with high internal consistency (Cronbach's α =0.87).

Work Ability was assessed using an adapted single-item measure derived from the Work Ability Index (2, 16). Participants were asked to compare their current work ability with their lifetime best (“Compare your current work ability with your best-ever work ability”), rated on a five-point scale ranging from 1 (*very poor*) to 5 (*excellent*).

Physical activity was assessed using a single item based on the WHO PA guidelines (14), recommending at least 150 minutes of moderate-intensity PA per week. Responses were dichotomized to indicate whether participants met the recommendation (0 = does not meet the guidelines; 1 = meets the guidelines).

### 2.3 Data collection procedure

Data were collected in December 2025 using a structured online questionnaire administered *via* Google Forms. A non-probability convenience sampling strategy was used. Recruitment was based on voluntary participation and relied on two complementary channels to reach early childhood education (ECE) teachers across Croatia. First, the survey link was distributed to kindergarten administrations with a request to share it with teachers through their internal communication systems. Second, the link was disseminated through professional teacher networks, whereby coordinators and heads of regional and local professional councils shared the invitation during or following professional meetings. Because the survey link was distributed through multiple administrative and professional channels, the total number of eligible ECE teachers who received the invitation could not be determined. Consequently, a response rate could not be calculated.

Participation was voluntary and anonymous. Before accessing the questionnaire, all potential participants were provided with written information about the study aims, data handling procedures, and their right to withdraw at any time without consequences. Completion of the questionnaire was taken to indicate informed consent.

### 2.4 Statistical analysis

All statistical analyses were performed using IBM SPSS Statistics 29. Descriptive statistics (mean and standard deviation) were calculated for all study variables. Distributional normality was evaluated through visual inspection of histograms and by examining skewness and kurtosis values, which were within acceptable ranges for parametric analyses. Internal consistency of the newly developed multi-item scales was assessed using Cronbach's α, and their factorial structure was examined through exploratory factor analysis (EFA). Sampling adequacy was evaluated using the Kaiser–Meyer–Olkin (KMO) measure, and the suitability of the correlation matrix for factor analysis was assessed with Bartlett's test of sphericity.

Linear associations between work ability, musculoskeletal pain, work-related functional limitations, and exposure to work-related stressors were analyzed using Pearson's correlation coefficient. Group differences according to compliance with WHO PA guidelines (14) were examined using independent-samples t-tests. Effect sizes were reported using Cohen's *d* (17).

The contribution of health and psychosocial variables to the explanation of work ability was examined using hierarchical multiple regression with two blocks of predictors. The first block included demographic variables (age, educational attainment, kindergarten location, and age group of children). The second block included health and psychosocial variables (compliance with WHO PA recommendations, work-related stressors, musculoskeletal pain, and work-related functional limitations). For each model, the total explained variance (*R*^2^) and the change in explained variance (Δ*R*^2^) were reported. Multicollinearity diagnostics indicated acceptable levels of tolerance and variance inflation factors (all tolerance values > 0.50 and all VIF values < 2.0), indicating no evidence of problematic multicollinearity.

Potential indirect associations were tested using two mediation analyses performed with the PROCESS macro for SPSS, Version 5.0, Model 4 (18). Indirect effects were estimated using bias-corrected bootstrapping with 5,000 resamples and 95% confidence intervals. The following variables were included as covariates in both mediation models: age, educational attainment, kindergarten location, age group of children, and compliance with WHO PA guidelines.

## 3 Results

Descriptive statistics and Pearson's correlations among the main study variables are presented in Table 2. The mean work ability score among ECE teachers was 4.13 (*SD* = 0.70). The mean musculoskeletal pain score was 2.48 (*SD* = 0.88), while work-related functional limitations and work-related stressors averaged 2.19 (*SD* = 0.99) and 2.97 (*SD* = 0.88), respectively. Work ability was significantly and negatively associated with musculoskeletal pain, work-related functional limitations, and work-related stressors. The strongest positive association was observed between musculoskeletal pain and work-related functional limitations (*r* = 0.655, *p* < 0.01). All remaining occupational health variables were positively intercorrelated and negatively associated with work ability. These findings support Hypothesis 1, indicating that higher levels of musculoskeletal pain, work-related functional limitations, and work-related stressors were associated with lower work ability among early childhood educators. Musculoskeletal pain was most frequently reported in the lower back (88.3%), followed by the neck (80.3%) and shoulders/upper limbs (75.8%).

**Table 2:** Descriptive statistics and inter-correlations among study variables (*N* = 472).

Variable	*M*	*SD*	1	2	3	4
1. Work ability	4.13	0.70	—			
2. Musculoskeletal pain	2.48	0.88	−0.113^*^	—		
3. Work-related functional limitations	2.19	0.99	−0.258^**^	0.655^**^	—	
4. Work-related stressors	2.97	0.88	−0.204^**^	0.404^**^	0.474^**^	—

^*^p < 0.05.

^**^p < 0.01.

A hierarchical regression analysis was conducted to examine factors associated with work ability (Table 3). In Model 1, which included demographic variables (age, educational attainment, kindergarten location, and age group of children), the model was not statistically significant (*R*^2^ = 0.016, *p* = 0.113). In Model 2, the addition of occupational health factors (musculoskeletal pain, work-related functional limitations, work-related stressors, and compliance with WHO physical activity guidelines) significantly improved the model (Δ*R*^2^ = 0.093, Δ*F*(4, 463) = 12.141, *p* < 0.001), with the final model explaining 10.9% of the variance in work ability (*R*^2^ = 0.109; Adjusted *R*^2^ = 0.094).

**Table 3:** Hierarchical regression analysis of factors associated with work ability among early childhood education teachers.

Predictor	B	SE	β	*p*
Model 1: Demographics (adjusted *R*^2^ = 0.007, *SE* = 0.694)				
Age (years)	0.005	0.003	0.076	0.104
Educational attainment	0.024	0.039	0.029	0.535
Kindergarten location	−0.047	0.075	−0.029	0.531
Age group of children	0.038	0.021	0.083	0.075
Model 2: Demographics + occupational health factors				
(adjusted *R*^2^ = 0.094, *SE* = 0.663)				
Age (years)	0.006	0.003	0.103	0.024
Educational attainment	0.042	0.038	0.050	0.267
Kindergarten location	−0.048	0.072	−0.030	0.503
Age group of children	0.031	0.021	0.068	0.135
Musculoskeletal pain	0.089	0.047	0.112	0.062
Work-related functional limitations	−0.190	0.043	−0.270	< 0.001
Work-related stressors	−0.101	0.041	−0.127	0.014
WHO PA Guidelines	0.099	0.063	0.070	0.118

Model 1: *F*_(4, 467)_ = 1.878, *p* = 0.113.

Model 2: *F*_(8, 463)_ = 7.099, *p* < 0.001; Δ*R*^2^ = 0.093; Δ*F*_(4, 463)_ = 12.141, *p* < 0.001.

In the final model, work-related functional limitations (β = −0.270, *p* < 0.001) and work-related stressors (β = −0.127, *p* = 0.014) were negatively associated with work ability. Age showed a small positive association with work ability (β =0.103, *p* =0.024). Musculoskeletal pain (β = 0.112, *p* = 0.062) and compliance with WHO physical activity guidelines (β = 0.070, *p* = 0.118) were not independently associated with work ability. These findings support Hypothesis 2, indicating that occupational health factors explained additional variance in work ability beyond demographic characteristics.

A comparison of early childhood teachers according to compliance with WHO physical activity guidelines showed that those who met the recommendations reported higher work ability, lower musculoskeletal pain, fewer work-related functional limitations, and lower exposure to work-related stressors than those who did not meet the guidelines (Table 4). All group differences were statistically significant (*p* ≤ 0.009). According to Cohen's criteria, the observed effect sizes were small (*d* = 0.25–0.36), supporting Hypothesis 3.

**Table 4:** Comparison of health indicators and work ability according to compliance with the WHO PA guidelines.

Variable	Does not meet WHO (*n* = 260)	Meets WHO (*n* = 212)	*P*	Cohen *d*
Work ability	4.06 (0.66)	4.23 (0.73)	0.009	0.25
Musculoskeletal pain	2.60 (0.80)	2.33 (0.94)	0.001	0.30
Work-related functional limitations	2.35 (1.02)	2.00 (0.92)	0.001	0.36
Work-related stressors	3.09 (0.87)	2.83 (0.87)	0.001	0.30

The mediation analysis (Table 5), controlling for age, educational attainment, kindergarten location, age group of children, and compliance with WHO PA guidelines, showed that higher levels of musculoskeletal pain were statistically associated with greater work-related functional limitations (*B* = 0.71; *p* = 0.001). Work-related functional limitations were statistically associated with lower work ability (*B* = −0.22; *p* = 0.001). When controlling for functional limitations, the association between musculoskeletal pain and work ability was not statistically significant (*B* = 0.07; *p* = 0.13), whereas the indirect association through functional limitations remained significant (*B* = −0.16, 95% *CI* [−0.23, −0.09]). Accordingly, Hypothesis 4 was supported at the level of statistical association.

**Table 5:** Mediation analysis: work-related functional limitations as a mediator of the relationship between musculoskeletal pain and work ability.

Path/relationship	*B*	*SE*	*p*	95% CI
Pain → Work-related Functional Limitations	0.713	0.041	0.001	[0.634, 0.793]
Work-related Functional Limitations → Work Ability	−0.223	0.042	0.001	[−0.305, −0.141]
Pain → Work Ability (direct effect)	0.071	0.047	0.133	[−0.022, 0.163]
Indirect effect (pain → limitations → work ability)	−0.159	0.036	–	[−0.234, −0.092]

All effects are adjusted for age, educational attainment, kindergarten location, age group of children, and compliance with WHO PA guidelines.

A second mediation model (Table 6), controlling for age, educational attainment, kindergarten location, age group of children, and compliance with WHO PA guidelines, examined work-related stressors as a potential mediator. Musculoskeletal pain was statistically associated with higher levels of work-related stressors (*B* = 0.41; *p* = 0.001), while work-related stressors were statistically associated with lower work ability (*B* = −0.16; *p* = 0.001). When controlling for stressors, the association between musculoskeletal pain and work ability was not statistically significant (*B* = −0.02; *p* = 0.55), whereas the indirect association through stressors remained significant (*B* = −0.06, 95% *CI* [−0.10, −0.03]). Compliance with WHO PA guidelines was associated with lower levels of work-related stressors (*B* = −0.16; *p* = 0.033), whereas working with older or mixed-age groups was associated with higher levels of work-related stressors (*B* = 0.06; *p* = 0.019). These results were consistent with Hypothesis 5 at the level of statistical association.

**Table 6:** Mediation analysis: work-related stressors as a mediator of the relationship between musculoskeletal pain and work ability.

Path/relationship	*B*	*SE*	*p*	95% CI
Pain → stressors	0.413	0.043	< 0.001	[0.328, 0.497]
Stressors → work ability	−0.156	0.040	< 0.001	[−0.234, −0.078]
Pain → work ability (direct effect)	−0.024	0.040	0.554	[−0.103, 0.056]
Indirect effect (pain → stressors → work ability)	−0.064	0.016	–	[−0.097, −0.033]

All effects are adjusted for age, educational attainment, kindergarten location, age group of children, and compliance with WHO PA guidelines.

## 4 Discussion

The prevalence of musculoskeletal pain symptoms among preschool teachers in the present study was high, particularly when all levels of symptom frequency, from rarely to daily, were considered. Lower back pain was reported by 88% of participants, neck pain by 80%, and shoulder and upper limb pain by 76%, while substantial prevalences were also observed for the knees (62%), feet (61%), and hips (54%). However, the prevalence of symptoms reported as often or daily was considerably lower; for example, 42.2% of participants reported lower back pain at these frequencies. This distinction is important because most previous studies have reported either 12-month prevalence or only moderate-to-frequent symptoms, limiting direct comparability. Differences in prevalence across studies are therefore expected and may reflect variations in prevalence definitions, recall periods, study populations, and working conditions. Although the core occupational tasks of preschool teachers are broadly similar, the frequency and duration of physically demanding activities, including lifting children, prolonged floor-level work, squatting, kneeling, and forward bending, may differ between settings because of differences in classroom organization, available equipment, and work practices. These differences may contribute to the variability in reported prevalence across studies. Nevertheless, the overall pattern observed in the present study is consistent with previous evidence demonstrating a high burden of musculoskeletal symptoms among teachers and early childhood educators. Erick and Smith (3) reported prevalence estimates ranging from 39% to 95% across teaching populations, while the recent meta-analysis by Tahernejad et al. (19) reported pooled prevalence estimates of 47% for neck and lower back pain and 44% for shoulder pain, with substantial between-study heterogeneity. Similarly, Converso et al. (20) found that 75.4% of preschool teachers experienced back pain. Despite differences in prevalence estimates, the lower back, neck, and shoulders consistently remained the most frequently affected body regions across studies, indicating that this pattern is closely related to the physical demands inherent in preschool teaching.

The high prevalence of musculoskeletal symptoms was accompanied by pronounced work-related functional limitations. Substantial proportions of participants reported at least moderate difficulties with bending, squatting, and lifting children or equipment, prolonged standing and walking, and maintaining energy during the workday. Difficulties were most prevalent for bending, squatting, and lifting, for which almost half of the participants reported at least moderate limitations. These findings are consistent with the physical demands of preschool teaching, where daily work involves repeated lifting and carrying of children, prolonged floor-level work, squatting, kneeling, and forward bending. Repeated exposure to these activities places considerable biomechanical demands on the spine and lower extremities and may contribute to musculoskeletal pain as well as difficulties in performing routine occupational tasks. This interpretation is supported by previous studies. Sottimano et al. (4) reported that 93.6% of preschool teachers lifted children daily and 61.1% frequently worked in a squatting position, with both activities being significantly associated with neck and lower back pain. Likewise, Turvill et al. (5) identified lifting children, stooping, working at low heights, and prolonged floor-level activities as common physical demands experienced by early childhood educators. The findings of the present study are consistent with this evidence and indicate that work-related functional limitations are closely associated with the repetitive biomechanical demands characteristic of preschool teaching.

Hierarchical regression analysis showed that demographic variables, including age, educational level, and kindergarten location, did not contribute substantially to the explanation of work ability, which is consistent with previous research suggesting that sociodemographic characteristics explain only a small proportion of variance in this construct (1, 21, 22). When occupational health variables were entered into the model, the explained variance increased significantly. Work-related functional limitations and work-related stressors emerged as the strongest negative correlates of work ability. These results are consistent with previous research highlighting the importance of both physical and psychosocial working conditions in educational and care professions. For example, Viotti et al. (10) reported a strong association between physical demands and work ability among kindergarten teachers and demonstrated that several psychosocial job resources buffered the negative effects of physical workload. Similarly, Nislin et al. (11) emphasized the importance of balancing job demands and resources in early childhood education settings and identified supervisor support as a key occupational resource associated with employee well-being and work functioning. Viewed alongside previous evidence, the present results support the interpretation that work ability is influenced more strongly by occupational health and workplace conditions than by demographic characteristics, which is consistent with the Job Demands–Resources framework. Nonetheless, the final model explained a relatively modest proportion of the variance in work ability (10.9%), suggesting that work ability is a multifactorial construct influenced by additional occupational, psychosocial, organizational, and individual factors beyond those included in the present study.

The mediation analyses indicated that musculoskeletal pain was not directly associated with work ability once work-related functional limitations and work-related stressors were taken into account. Instead, musculoskeletal pain was statistically linked to greater functional limitations and higher levels of work-related stressors, both of which were associated with lower work ability. Within the tested models, this pattern suggests that the statistical association between musculoskeletal pain and work ability may be embedded within broader occupational processes rather than reflecting a direct association alone. At the same time, the cross-sectional design does not allow conclusions regarding temporal ordering, and alternative explanations remain plausible, including the possibility that lower work ability influences the perception of pain and work-related difficulties.

Several mechanisms may help explain these associations. Everyday work in early childhood education involves sustained physical exertion and repetitive postural loading associated with routine childcare tasks. Such cumulative biomechanical exposure may contribute to tissue fatigue and the gradual development of musculoskeletal symptoms. Evidence from longitudinal studies has identified heavy physical work, repetitive movements, awkward postures, and lifting tasks as important risk factors for work-related musculoskeletal disorders (23). More recently, Abreek-Sarhan et al. (24) reported that awkward and static postures, repetitive motions, and inadequately designed equipment were significantly associated with musculoskeletal disorders among preschool educators. A related study from the same research group further showed that professional seniority, independent of age, was associated with higher odds of neck, hand/wrist, and lower back pain, supporting the notion that cumulative occupational exposure may contribute to symptom development over time (6).

The prominence of work-related stressors in the present models suggests that musculoskeletal symptoms may not be understood solely in biomechanical terms. This interpretation is supported by previous research. Shi et al. (9) found that low social support predicted the persistence of low back pain among daycare workers over a one-year period. Likewise, Eatough et al. (12) demonstrated that psychosocial work stressors were associated with musculoskeletal symptoms through increased psychological strain. In a longitudinal study of healthcare workers, Vieira et al. (13) reported that stress and burnout were related to multisite musculoskeletal pain and that part of this association was statistically explained by sleep quality. Similarly, Mateos-González et al. (25) showed that psychosocial factors, including job demands and social support, mediated the associations between occupational risk factors and musculoskeletal symptoms among nursing aides. Additional evidence from non-teaching occupations indicates that occupational stress may be indirectly associated with work-related musculoskeletal disorders through sleep-related mechanisms (26). Viewed alongside this body of evidence, the present results suggest that the statistical association between musculoskeletal pain and work ability may reflect the combined influence of physical and psychosocial job demands. Within this framework, musculoskeletal pain may be viewed as an indicator of physical strain, whereas functional limitations and work-related stressors represent broader occupational demands. Given the cross-sectional design, no temporal or causal interpretations can be drawn.

PA was not independently associated with work ability in the regression model. Nevertheless, physically active preschool teachers reported lower levels of musculoskeletal pain, lower exposure to work-related stressors, and fewer work-related functional limitations. This pattern is broadly consistent with previous evidence suggesting that regular PA is associated with better physical and occupational health outcomes (27, 28). From a theoretical perspective, PA may be viewed as an individual health resource that supports adaptation to both physical and psychosocial job demands. Within the Job Demands–Resources framework, personal resources are considered important factors that may enhance resilience and facilitate coping with occupational challenges. Similarly, biopsychosocial models of musculoskeletal disorders emphasize the role of physical fitness and general health in maintaining functional capacity. Research conducted in educational settings has also shown that higher habitual PA tends to co-occur with better postural control and lower physical strain (29). Although the present findings do not support a direct association between PA and work ability, they suggest that PA is statistically linked to a more favorable health and occupational profile among preschool teachers. Given the cross-sectional design, the direction of these associations remains uncertain.

This study has several limitations. The cross-sectional design precludes conclusions about temporal ordering, and the use of an online questionnaire introduces the possibility of self-selection bias. The possibility of a healthy worker effect should also be considered, particularly given the voluntary online survey design. Teachers experiencing severe health problems, prolonged sickness absence, or reduced work participation may have been less likely to participate, potentially resulting in an underestimation of occupational health problems. Furthermore, a non-probability convenience sampling approach was used, and because the survey link was distributed through multiple administrative and professional channels, the total number of eligible ECE teachers who received the invitation could not be determined. This limitation precluded the calculation of a response rate and restricted the evaluation of sample representativeness. The sample was highly feminized, reflecting the composition of the profession, which limits generalizability to male educators. Although the study-specific scales demonstrated satisfactory internal consistency, further psychometric evaluation is required, including confirmatory factor analysis, test-retest reliability, and validation in independent samples. In particular, the Health Burden measure combined disease presence and perceived functional impact into a single ordinal response for each condition. Although its role in the primary analyses was limited, future studies should assess disease status and functional impact as separate constructs using independently validated measures. PA and work ability were assessed using single-item measures, which are pragmatic tools but offer limited precision and do not capture different domains or intensities of activity, nor the multidimensional structure of work ability. Associations involving these variables should therefore be interpreted with appropriate caution. Moreover, the study did not assess potentially relevant psychological factors such as burnout, depressive symptoms, or other indicators of mental health, which may influence both work ability and occupational stress. Finally, the study design does not allow determination of the directionality between musculoskeletal pain and work-related functional limitations; pain may reduce functional capacity, whereas functional strain may increase pain symptoms, and both processes may operate concurrently. Longitudinal research is needed to disentangle these bidirectional pathways.

## 5 Practical Implications

The present results suggest several practical considerations for supporting work ability among preschool teachers. The strong associations observed between work-related functional limitations and lower work ability highlight the potential value of reviewing the biomechanical demands of everyday work tasks and providing education on safe movement strategies, load management, and recovery during the working day. Incorporating basic ergonomic principles into both initial teacher education and continuing professional development may help educators better manage the physical demands of their occupation. The mediation analyses further indicated that musculoskeletal pain was statistically linked to work ability through functional limitations and work-related stressors, suggesting that workplace initiatives aimed at maintaining functional capacity and reducing physical strain may be beneficial. Although causal conclusions cannot be drawn from the present data, preschool teachers who met WHO PA recommendations reported more favorable health and occupational characteristics, supporting the inclusion of PA within broader workplace health promotion initiatives. The associations observed between work-related stressors and work ability also point to the importance of organizational factors. Issues such as time pressure, workload distribution, group size, and the availability of collegial and supervisory support may represent potential targets for intervention. Addressing these challenges will likely require collaboration among educational practitioners, administrators, ergonomists, kinesiologists, and occupational health professionals in order to develop feasible approaches that acknowledge both the physical and psychosocial demands of early childhood education work.

## 6 Conclusion

This study examined multiple health and psychosocial factors associated with work ability among preschool teachers by jointly analyzing musculoskeletal pain, work-related functional limitations, work-related stressors, and PA. The results suggest that the statistical relationship between musculoskeletal pain and work ability may operate through work-related functional limitations and work-related stressors rather than through a direct association alone. PA was statistically related to more favorable health indicators, although no direct association with work ability was observed. The findings highlight the relevance of integrating physical and psychosocial aspects when examining work ability in early childhood education settings. The study also provides initial psychometric evidence for the measures used, offering a starting point for the further development and validation of instruments tailored to the Croatian context.

## Data Availability

The original contributions presented in the study are included in the article/Supplementary material, further inquiries can be directed to the corresponding author.
