# Supplementary material for: Occupational health factors associated with work ability among early childhood education teachers
Source: Front Public Health. 2026 Sep 1;14:1847583. doi: 10.3389/fpubh.2026.1847583 (PMC13574984; doi:10.3389/fpubh.2026.1847583)
Supplement: Supplementary file 1 [file Data_Sheet_1.pdf]

Supplementary Material

1 Supplementary Data

Table S1. Health Burden (HB) items and prevalence of chronic health conditions (N = 472)

Question: Please indicate whether you have any of the following diagnosed health conditions and the extent to which they affect your daily life and work.

Health problem	Not present (1)	Present, no impact (2)	Mild impact (3)	Moderate impact (4)	Severe impact (5)
Hypertension	84.7%	6.8%	4.7%	2.3%	1.5%
Cardiovascular diseases	86.9%	6.1%	4.0%	1.7%	1.3%
Chronic respiratory diseases (e.g., asthma)	84.7%	5.1%	4.9%	3.4%	1.9%
Thyroid diseases	79.7%	7.8%	5.9%	3.2%	3.4%
Osteoporosis	90.7%	3.2%	2.8%	1.7%	1.7%
Arthritis or other rheumatic diseases	84.7%	5.9%	4.0%	3.4%	1.9%
Spinal diseases (e.g., herniated disc, spondylosis)	65.0%	8.7%	10.0%	8.7%	7.6%
Other health condition	66.7%	10.0%	12.7%	5.9%	4.7%

Table S2. Musculoskeletal Pain (MSP) scale items and prevalence by body region in the last six months (N = 472)

Question: How often have you experienced musculoskeletal pain during the past six months?

Body region	Never (1)	Rarely (2)	Occasionally (3)	Often (4)	Daily (5)
Neck	19.7%	22.0%	34.1%	16.3%	7.8%
Shoulders and arms	24.2%	24.8%	28.4%	15.9%	6.8%
Lower back	11.7%	14.0%	32.2%	26.1%	16.1%
Hips	45.6%	22.9%	15.9%	11.3%	4.2%
Knees	37.9%	23.3%	21.0%	11.2%	6.6%
Feet	39.5%	27.6%	20.2%	6.8%	5.9%

Table S3. Work-related Functional Limitations (WFL) scale items and prevalence in the last month (N = 472)

Question: To what extent do your health problems affect the following aspects of your work?

Work task	Not at all (1)	Slightly (2)	Moderately (3)	Significantly (4)	Extremely (5)
Participation in activities with children	49.6%	23.6%	17.7%	6.0%	3.2%
Prolonged standing and walking	34.7%	29.4%	19.3%	11.7%	4.9%
Bending, squatting, and lifting children or equipment	28.8%	22.7%	23.3%	15.3%	10.0%
Concentration and attention at work	49.8%	26.7%	16.1%	5.1%	2.3%
Overall energy level and resistance to fatigue	26.1%	30.7%	23.3%	12.3%	7.6%
Performing physically demanding pedagogical activities	36.0%	28.2%	22.0%	8.1%	5.7%

Table S4. Work-related Stressors (WRS) scale items and prevalence in the last few months (N = 472)

Question: How often have you experienced stress related to the following aspects of your work during the past few months?

Stressor	Never (1)	Rarely (2)	Occasionally (3)	Often (4)	Very often (5)
Too many children in the group	12.1%	13.6%	28.8%	19.9%	25.6%
Lack of time to complete all tasks	4.9%	16.1%	32.0%	25.8%	21.2%
Pressure from administrative demands	10.8%	20.6%	31.4%	18.0%	19.3%
Working with children with special needs	17.8%	20.3%	23.9%	19.9%	18.0%
Relationships with parents	13.6%	29.2%	28.8%	16.9%	11.4%
Relationships with colleagues	23.3%	36.2%	23.9%	9.3%	7.2%
Lack of support from superiors	24.4%	28.0%	20.1%	12.9%	14.6%
Unpredictable situations at work	8.5%	25.8%	35.8%	16.1%	13.8%

Lack of resources (space, materials)	17.4%	23.1%	25.2%	16.7%	17.6%

Table S5. Item-level psychometric characteristics of the MSP, WFL, and WRS scales

Scale/Factor	Items	Communalities	Factor Loadings
MSP	6	0.46–0.57	0.68–0.76
WFL	6	0.66–0.80	0.81–0.89
WRS	9	0.39–0.61	0.63–0.78

Note. MSP = Musculoskeletal Pain; WFL = Work-related Functional Limitations; WRS = Work-related Stressors.
